# Continued Implementation and Use of a Digital Informal Care Support Platform Before and After COVID-19: Multimethod Study

**DOI:** 10.2196/54734

**Published:** 2024-12-31

**Authors:** Nikita Sharma, Christian Wrede, Sofia Bastoni, Annemarie Braakman-Jansen, Lisette van Gemert-Pijnen

**Affiliations:** 1 Faculty of Behavioural, Management and Social Sciences, University of Twente, Drienerlolaan 5, Enschede, 7522 NB, Netherlands, 31 053 489 9111; 2 Faculty of Information Technology and Electrical Engineering, University of Oulu, Oulu, Finland

**Keywords:** digital care platform, eHealth, implementation, informal care, new digital normal, COVID, Caren, consolidated framework

## Abstract

**Background:**

With the growing need of support for informal caregivers (ICs) and care recipients (CRs) during COVID-19, the uptake of digital care collaboration platforms such as Caren increased. Caren is a platform designed to (1) improve communication and coordination between ICs and health care professionals, (2) provide a better overview of the care process, and (3) enhance safe information sharing within the care network. Insights on the impact of COVID-19 on the implementation and use of informal care platforms such as Caren are still lacking.

**Objective:**

This study aimed to (1) identify technology developers’ lessons learned from the continued implementation of Caren during COVID-19 and (2) examine pre-post COVID-19 changes in usage behavior and support functionality use of Caren.

**Methods:**

A focus group with developers of the Caren platform (N=3) was conducted to extract implementation lessons learned. Focus group data were first analyzed deductively, using the Consolidated Framework for Implementation Research domains (ie, individual characteristics, intervention characteristics, inner setting, and outer setting). Later, inductive analysis of overarching themes was performed. Furthermore, survey data were collected in 2019 (N=11,635) and 2022 (N=5573) among Caren platform users for comparing usage behavior and support functionality use. Data were analyzed using descriptive and inferential statistics.

**Results:**

Several lessons from the continued implementation of Caren during COVID-19 were identified. Those included, for example, alternative ways to engage with end users, incorporating automated user support and large-scale communication features, considering the fluctuation of user groups, and addressing data transparency concerns in health care. Quantitative results showed that the number of ICs and CRs who used Caren several times per day increased significantly (*P*<.001 for ICs and CRs) between 2019 (ICs: 23.8%; CRs: 23.2%) and 2022 (ICs: 35.2%; CRs: 37%), as well as the use of certain support functionalities such as a digital agenda to make and view appointments, a messaging function to receive updates and communicate with formal and informal caregivers, and digital notes to store important information.

**Conclusions:**

Our study offers insights into the influence of the COVID-19 pandemic on the usage and implementation of the digital informal care support platform Caren. The study shows how platform developers maintained the implementation during COVID-19 and which support functionalities gained relevance among ICs and CRs throughout the pandemic. The findings can be used to improve the design and implementation of current and future digital platforms to support informal care toward the “new digital normal.”

## Introduction

The proportion of older adults in the global population is rapidly rising. According to the 2019 United Nations projections [1], the number of individuals older than 65 years is expected to nearly double by 2050, with Europe and North America seeing an increase from 5% to 16% of their total population. As life expectancy continues to rise, so does the likelihood of developing chronic diseases, which can impede a person’s ability to live independently and necessitate ongoing care.

While the demand for formal care and support is expected to rise, it is important to acknowledge the often overlooked role of informal care, which provides a silent but significant form of support for individuals in need. Informal caregivers (ICs) play a critical role in providing unpaid and ongoing care to chronically ill, frail, or otherwise care-dependent individuals [2]. They assist their spouses, parents, family members, friends, or neighbors with a variety of daily tasks, such as shopping, personal care, household chores, and administrative duties. Furthermore, ICs provide emotional and practical support, including assistance with medical treatments [2,3]. Although informal care enables care recipients (CRs) to remain in their homes for longer, it can place significant strain on ICs [4], adversely affecting their physical, social, and emotional well-being [5-7]. The COVID-19 pandemic has likely exacerbated the challenges faced by this already vulnerable group [8], leading to increased stress and burden for caregivers [9].

One potential solution to address these challenges and alleviate caregiver burden is through the use of technological tools. In fact, a substantial body of evidence suggests that eHealth tools can be cost-effective [10,11] for improving ICs’ well-being and facilitating care at a distance [12]. eHealth can be defined as “the use of technology to improve health, well-being, and healthcare” [13]. Several types of eHealth solutions are available for ICs. These solutions are either used by ICs and CRs alone or meant for joint use [14]. Devices aimed at facilitating communication among ICs, CRs, and formal caregivers are of growing importance to optimizing the care process and supporting care management [1]. Those technologies are in line with recent paradigms such as “Connected Health.” Connected Health is “built around the patient’s needs, and health-related data is shared in such a way that the patient can receive care in the most proactive and efficient manner possible. All stakeholders in the process are ‘connected’ by means of timely sharing and presentation of accurate and pertinent information regarding patient status through smarter use of data, devices, communication platforms and people*”* [15].

An example of connected health in the Netherlands is Caren. Caren is a platform designed to (1) improve communication and coordination between health care professionals and ICs, (2) provide a better overview of the care process, and (3) enhance safe information sharing within the care network [16,17]. The platform is free to use for ICs and CRs, counting approximately 600,000 active users and 9000 affiliated health care providers (as of September 2024) [17]. The platform offers four main functions: (1) a shared agenda to make and view appointments (calendar), (2) a messaging function to receive updates and communicate with formal and informal caregivers, (3) a digital storage for important information such as names and addresses, and (if applicable) (4) a connection to the electronic client record of the involved home care provider (dossier).

Digitalization of formal and informal care was already growing before COVID-19 [18], but facing the pandemic, the use of digital informal care platforms such as Caren was a promising resource.

However, insight into possible changes in usage behavior of ICs and CRs pre- and postpandemic, as well as lessons learned for continued implementation, remains limited. These insights could help enhance the design and implementation of digital care platforms, improving their effectiveness in supporting informal care within the evolving landscape of the “new digital normal.” Therefore, the objective of this study is 2-fold: (1) to identify lessons learned about the continued implementation of Caren throughout the COVID-19 pandemic from the technology developers’ perspective and (2) to conduct a pre-post COVID-19 comparison of usage behavior and support functionality use of Caren.

## Methods

### Research Design

This study applied a multimethod design including qualitative and quantitative data collection. To obtain lessons learned from the continued implementation of Caren, a focus group was conducted with the development team of Caren. To conduct a pre-post COVID-19 comparison of usage behavior, a cross-sectional web-based survey was conducted among ICs and CRs who were users of the platform Caren pre-COVID (2019) and post onset (2022).

### Ethical Considerations

The ethics committee of the University of Twente (Faculty of Behavioural, Management and Social Sciences) provided ethics approval for this study according to European regulations (request numbers: 220250, 221180, and 190190). The surveys were designed to ensure complete anonymity, with no personal data being collected from participants. For the focus groups, participant identities were de-identified to maintain confidentiality and protect their privacy.All participants provided informed consent prior to participation in the study.

### Sampling Procedure

Participants eligible for the focus group were required to be at least 18 years of age and hold a position in the development team of the Caren platform. Recruitment took place via corporate email, supplying information about the purpose and content of the focus group on the basis of which participants could indicate their interest to take part. Each of the approached potential participants agreed to participate in the focus group which took place in-person at the developers’ company.

Participants for the surveys were recruited through purposive sampling. In 2019 and 2022, a cross-sectional web-based survey was offered to users of the platform Caren via a pop-up message on the platform. Participants were included in the study if, at the time of data collection, they used the platform either as an IC or as a CR. Responses from subjects younger than 18 years and incomplete responses, deemed as where the respondent did not provide answers to all mandatory questions, were excluded from the analysis.

### Data Collection

#### Focus Group

We created a focus group guide (Multimedia Appendix 1) containing open-ended questions aimed at identifying lessons learned from the continued implementation of Caren throughout the pandemic from the technology developers’ perspective. The focus group consisted of three parts: (1) impacts of COVID-19 on the Caren platform, (2) concrete reactions to those impacts, and (3) lessons learned by the Caren development team throughout the pandemic. All parts were structured according to the Consolidated Framework for Implementation Research (CFIR) [19]. Accordingly, within all parts, aspects related to four domains of the CFIR were addressed, including (1) *characteristics of individuals* (users of the Caren platform), (2) *intervention characteristics* (the Caren platform), (3) *inner setting* (the implementing organization meaning those who develop and implement Caren), and (4) *outer setting* (the broader implementation context, ie, health care setting of Caren). Note that the fifth domain, “process,” was not explicitly addressed in this study due to the implementation of the Caren platform being already completed. However, according to the CFIR guidelines, activities occurring after the initial implementation process are considered part of the innovation itself and are categorized within the *intervention characteristics* domain and the *inner setting* domain [20]. The focus group session was audio-recorded and lasted 1.5 hours.

#### Web-Based Survey

The web-based survey (MultimediaAppendices 2  3) was developed by a multidisciplinary group consisting of researchers in the field of health technology and technology designers. Relevant questions for the survey were formulated based on the research questions tailored to the support functionalities of Caren. The survey was pretested among the target group to collect feedback on the clarity of questions and adjustments to the wording were made accordingly before the survey was sent out for data collection in 2019 and 2022. This study reports findings on the following variables:

- Demographics: Participant characteristics including user role (IC or CR), age, gender, and housing situation.
- Caren platform usage:
  - Frequency of use (ranging from monthly to several times a day).
  - Usage of the different support functionalities of the platform (yes or no) including (1) a shared agenda to make and view appointments, (2) a messaging function to receive updates and communicate with formal and informal caregivers, (3) digital notes to store important information such as addresses and names, and (if applicable) (4) a connection to the electronic client record of the involved home care provider (dossier).

### Data Analysis

#### Qualitative Analysis

The audio-recording of the focus group session was transcribed verbatim and content analysis was performed using ATLAS.ti (version 8; ATLAS.ti Scientific Software). First, fragments that hold relevant information related to lessons learned from the continued implementation of Caren throughout the pandemic were selected and categorized into one of the 4 CFIR domains (characteristics of individuals, intervention characteristics, inner setting, and outer setting). Subsequently, selected fragments were further categorized inductively into overarching topics. To minimize single-researcher bias, 3 researchers (SB, NS, and CW) independently coded one-third of the data. A meeting took place to determine whether the codes described were proper interpretations of the data. The final coding scheme was defined on the basis of consensus between all researchers (SB, NS, and CW).

#### Quantitative Analysis

To enable comparison between 2019 and 2022, most of the variables from the original surveys needed to be recoded. For example, the answer categories for “frequency of use of Caren” in 2019 were reverse coded to match those of the same variable in 2022 (1 [monthly] to 6 [several times per day]). The data were analyzed using SPSS statistical software (version 25; IBM Corp). Descriptive statistics (means and SDs) were computed for continuous variables (age). Frequency distributions (n, %) were created for categorical variables (gender, housing situation, frequency of use of the platform, and usage of different support functions).

Kolmogorov-Smirnov tests were conducted on all continuous variables to test for normality. Nonparametric tests were used in case of not normally distributed data. Differences in demographic characteristics of ICs between 2019 and 2022 were tested using chi-square tests for categorical variables and Mann-Whitney *U* tests for continuous variables. In the same way, differences in demographics between 2019 and 2022 were tested for CRs.

Differences between 2019 and 2022 regarding ICs’ frequency of use of the platform were tested by chi-square tests. In the same way, differences regarding CRs’ frequency of use of the platform were tested. Generally, statistical significance was set at α<.05.

## Results

### Focus Group Results

In the following section, we present lessons learned from the continued implementation of Caren throughout the COVID-19 pandemic from the technology developer’s perspective. The lessons are grouped according to the 4 CFIR domains and are also available in tabular form in Multimedia Appendix 4.

#### Inner Setting

Within the domain *inner setting*, we identified 2 lessons learned that related to the implementing organization.

##### Act Promptly to Maintain Workflow

Amidst the COVID-19 pandemic, Caren’s team experienced escalated pressure in addressing user support. Although the type of help requests from users did not deviate significantly from those received before the pandemic (“more of the same”), the sheer volume of such requests surpassed the support team’s capacity. Consequently, there was an urgent need to recruit extra personnel. Moreover, the pandemic compelled the entire Caren team to operate remotely for the first time. To ensure the seamless flow of operations, it was crucial to strike a balance between personal and work life.

##### Find Innovative Solutions to Engage the Target Audience

The COVID-19 pandemic presented the Caren team with obstacles in their engagement with the target population, given that physical visits to end users and health care organizations were limited. While video conferencing was available as an alternative means of involving the target group in the development process, it was not always practical, particularly for digitally inexperienced people. To address this issue, the team devised innovative solutions, such as affixing a basic iPad to a mobile robot stationed in care institutions, to establish an interactive communication channel.

### Intervention Characteristics

Within the domain *intervention characteristics*, 3 lessons learned related to the changes in certain (technical) characteristics of the platform or services surrounding the platform were identified.

#### Automated User Support Is Essential

Due to an increasing number of platform users during the pandemic, automated user support became essential. In response to that, a chatbot functionality was implemented to provide automated and efficient assistance to customers (both ICs and care organizations) in resolving their issues.

#### Large-Scale Communication Features Are Valuable

With sudden changes in government regulations due to the fluctuating infection rates, care homes needed to adapt to the new guidelines with immediate effect. They were responsible for communicating the current guidelines and their consequences on the in-person visits between clients and their caregivers but had limited means to do so. Therefore, the functionality to send bulk messages was embedded within the Caren platform to quickly broadcast messages to all caregivers and patients at once.

#### Integrate Care Information Into a Single Platform

Due to COVID-19, ICs’ need for digital access to reliable care-related information became even more important. However, often care information is scattered across different care organizations and not accessible via one platform. To facilitate more integrated care, the Caren team plans to provide possibilities for integrating care information stemming from different electronic client records (such as radiographies, medical history, and blood test reports) in the platform.

### Characteristics of Individuals

Within the domain *characteristics of individuals*, we identified 2 lessons learned related to the end users of Caren throughout the COVID-19 pandemic.

#### Social Isolation Has a Positive Impact on the Adoption of Digital Technology

Due to COVID-19, a surge in the number of new Caren users was observed. This could be because the caregivers were not able to visit their loved ones personally (ie, social isolation), thus leaving not many options for them to connect to their loved ones. In this sense, the pandemic acted as a change catalyst, and caregivers went from “hesitant” to “forced” to use digital care tools such as Caren (as expressed by the technology developers).

#### Mind the Fluctuation of User Groups

Unpredictable, large-scale events, such as pandemics, can impact the user pool of digital care technologies and might result in dramatic changes in their composition. Overall the number of users increased; however, it impacted the sociodemographics of the user groups (eg, with the passing of numerous users belonging to vulnerable groups by infection or by acquiring new user groups). Specifically, mental health care organizations started adopting Caren during the pandemic, causing not only a change in the user group of Caren in terms of numerosity or sociodemographic characteristics but also expanding their entire line of business (eg, by becoming more “Business to Business”).

### Outer Setting

Within the domain *outer setting*, we identified 2 lessons learned related to the broader implementation context of Caren.

#### Invest in a Self-Sufficient and Sustainable Business Model

During COVID-19, new digital care platforms subsidized by the Dutch government emerged on the market. This competition was accelerated by a new governmental policy that obliged care institutions to provide clients insight into their own care data on the web. However, most of those new platforms lacked sustainable business models and were discontinued after the government funding ran out. This strengthened the Caren team in their choice of a business model that is independent from governmental funding and robust against rapid market change. Specifically, Caren relies now on a business-to-business-to-consumer model where health care institutions purchase an electronic client record that offers Caren as an add-on.

#### Address Concerns About Data Transparency in Health Care

COVID-19 accelerated the collection of care-related data on the web. However, participants reported that the fear of data transparency in health care can form a significant barrier to implementing platforms such as Caren. Although required by Dutch law, health care organizations are sometimes hesitant to be fully transparent about and share client data collected, as they fear that it could make it easier to trace errors back to individual caregivers. To address this, technology developers emphasized the importance of educating stakeholders about the benefits of digital data exchange within a client’s care network, such as an increased quality of care.

### Survey Results

#### Sample Characteristics

As can be seen in Table 1, the survey resulted in 11,635 completed responses from ICs in 2019 and 5573 responses in 2022. On average, ICs were older than 58 years (mean 58.7, SD 9.2 years in 2019 and mean 60.3, SD 9.3 years in 2022). The majority of ICs were female (9151/11,635, 78.7% in 2019; 4090/5573, 73.4% in 2022), living at a distance from their CR (10,830/11,635, 93% in 2019; 5224/5573, 95% in 2022), and reported a low care load (5951/11,635, 51.1% in 2019; 4200/5573, 75.4% in 2022). Among CRs, the survey resulted in 2352 completed responses in 2019 and 1285 responses in 2022. CRs were, on average, older than 64 years (mean 64.9, SD 14.2 years in 2019 and mean 66.1, SD 14.5 years in 2022), with the majority of them being female (1278/2352, 54.5% in 2019; 66/1285, 51.3% in 2022).

**Table 1.** Characteristics of both ICs and CRs in 2019 (T0) and 2022 (T1).

Variable		ICs^a^		CRs^b^	
		2019 (T0)	2022 (T1)	2019 (T0)	2022 (T1)
Total, n (%)		11,635 (83.2)	5573 (81.3)	2352 (16.8)	1285 (18.7)
Age (years), mean (SD)		58.7 (9.2)	60.3 (9.3)	64.9 (14.2)	66.1 (14.5)
Sex, n (%)					
	Female	9151 (78.7)	4090 (73.4)	1278 (54.5)	660 (51.3)
	Male	2443 (21)	1446 (26.3)	1068 (45.4)	620 (48.2)
	Other	41 (0.3)	17 (0.3)	6 (0.3)	5 (0.3)
Housing situation, n (%)					
	Living at a distance from CR	10,830 (93)	5224 (95)	N/A^c^	N/A
	Living together with CR	805 (7)	258 (5)	N/A	N/A
Care load, n (%)					
	Low (<8 hours per week)	5951 (51.1)	4200 (75.4)	N/A	N/A
	Medium (8‐24 hours per week)	4544 (39.1)	10,12 (18.2)	N/A	N/A
	High (>24 hours per week)	1140 (9.8)	361 (6.5)	N/A	N/A

a ICs: informal caregivers.

b CRs: care recipients.

c N/A: not applicable.

We found significant differences in sample characteristics between 2019 and 2022. ICs in 2022 were significantly older (*P*<.001), significantly more often male (*P*<.001), significantly more often living at a distance from their CR (*P*<.001), and reported significantly more often a low care load while significantly less often a medium or high care load (*P*<.05). CRs, on the other hand, were significantly older in 2022 than in 2019 (*P*<.001). CRs in 2019 and 2022 did not significantly differ regarding gender.

#### Overall Usage of Caren in 2019 and 2022 by ICs and CRs

**Figure 1.**
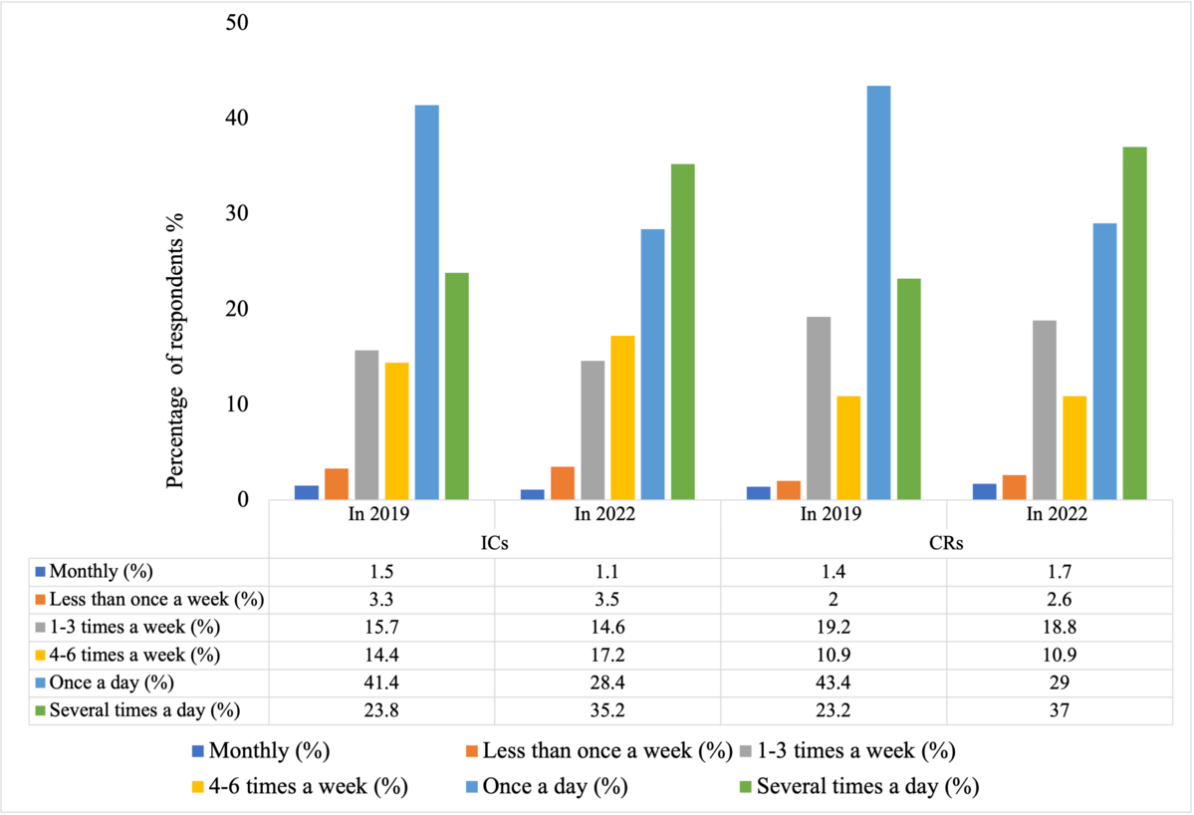
Frequency of use of Caren in 2019 and 2022 among informal caregivers and care recipients.CR: care recipient; IR: informal caregiver.

As displayed in Figure 1, descriptive statistics show that most ICs and CRs in 2019 and 2022 used the platform Caren at least once a day. Between 2019 and 2022, an increase of ICs and CRs is visible who used the platform several times a day.

We found significant differences in the frequency of use of Caren for ICs between 2019 and 2022 (*χ*^2^_5_=1868.390; *P*<.001). Specifically, ICs in 2022 were significantly more often using Caren several times a day than ICs from 2019. However, they were significantly less often using Caren monthly, 1‐3 times a week, or once a day.

We also found significant differences in the frequency of use of Caren for CRs between 2019 and 2022 (*χ*^2^_5_=484.159; *P*<.001). Specifically, CRs in 2022 were significantly more often using Caren several times per day than those in 2019. However, they were using Caren significantly less often 1‐3 times a week or once a day.

#### Usage of Support Functionalities

Table 2 shows that, in 2019, the most used platform functionality among ICs was a connection to the electronic client record of their CRs’ home care provider (“Dossier,” 84.9%), whereas in 2022, a digital notes function to store important information such as addresses and names was used most (“Notes,” 98.6%). Among CRs, a shared agenda to make and view appointments was used most in 2019 (“Calendar,” 70.2%), whereas in 2022 the digital notes function became the most used functionality (“Notes,” 90.3%).

**Table 2.** Usage of Caren’s support functionalities by both ICs and CRs in 2019 and 2022.

Support functionality and usage		ICs^a^		CRs^b^	
		2019 (T0)	2022 (T1)	2019 (T0)	2022 (T1)
Calendar: shared agenda to make and view appointments, %					
	Yes	48.8	68.1	70.2	89.2
	No	51.1	31.9	29.8	10.8
Messages: messaging function to receive updates and communicate with formal and informal caregivers, %					
	Yes	63.9	91.1	35.4	79.9
	No	36.1	8.9	64.6	20.1
Notes: digital notes to store important information such as addresses and names, %					
	Yes	22.6	98.6	18.6	90.3
	No	77.4	1.4	81.4	9.7
Dossier: a connection to the electronic client record of the involved home care provider, %					
	Yes	84.9	53.8	57.9	60.8
	No	15.1	46.2	42.1	39.2

a ICs: informal caregivers.

b CRs: care recipients.

Generally, the descriptives of Table 2 show an increase in the use of the calendar, messages, and notes functionalities among ICs between 2019 and 2022, while the use of the dossier function decreased. Among CRs, an increase in the use of all platform functionalities could be observed between 2019 and 2022.

## Discussion

### Principal Findings

The study identified several lessons that were learned about the continued implementation of the informal care platform Caren throughout the COVID-19 pandemic from the technology developers’ perspective. Furthermore, the study revealed pre-post COVID-19 changes in usage behavior and support functionality use among ICs and CRs.

Lessons learned relating to the continued implementation of Caren were categorized on the basis of the CFIR. The first domain is the *inner setting*, which refers to the environment of an organization or system where a particular health care intervention or program is being implemented [19]. The increasing use of digital technology during COVID-19 has impacted the work cultures in a multitude of ways. This includes organization of the work within teams, performance or measurement of work done, effective communication, and collaboration within or across teams [21]. The findings of this study are along similar lines. The technology developers and designers had to adjust to and accommodate new ways of working (eg, working from home). Broadly, these changes rely heavily on the usage of video- or audioconferencing tools, thus demanding organizations to upgrade their technology infrastructure [21]. However, in Caren’s case, the company had the infrastructure to manage IT overloads, which streamlined the process. Furthermore, as Caren employees have adapted to remote work, there is a growing tendency for organizations to maintain this practice.

The *outer setting* is the broader context in which an organization operates, encompassing the broader social, economic, and political factors that can impact the implementation of a health care intervention or program [19]. A direct impact of the used business model by organizations responsible for developing and facilitating care infrastructure (outer setting) became apparent in this study. Legislations were passed to take digital initiatives during the start of the pandemic, but these were not sustainable for the long term [22]. In the study by Bokolo [23], the impact of the shortage of government funding and reimbursement model was found to be significantly impacting the implementation of digital health tools during the COVID-19 pandemic. In that regard, Caren not depending on government funding facilitated its continued implementation. Finally, due to the increased use of technological interventions in care, enormous amounts of data on CRs are available. While artificial intelligence can help in improving the quality of in-general health care (by correct prediction, classification, and useful recommendations) [24,25], it is also mandatory to invest and implement new data security ways to ensure safety and privacy of such personal datasets [26]. In this study, technology developers and designers were also concerned regarding the transparency of the medical data.

Regarding the domain *intervention characteristics*, developers recommended relying on automated response functions. This is in line with the work of Zand and colleagues [27]. In fact, their analyses of electronic dialog data showed that patient-provider communication through automatic messaging can dramatically optimize workflows. In fact, allocating simple tasks to the chatbot leaves more time available for better patient care. Furthermore, chatbots are continuously available and have no waiting time. Aside from simple tasks, such as managing appointments or delivering bulk messages such as in the case of Caren, the authors suggest that chatbots can also be used for more sophisticated, although instrumental, parts of care.

Finally, regarding the domain of *characteristics of individuals*, developers indicated that the COVID-19 pandemic, and especially the social isolation measures that came from it, was a catalyst for the adoption of technology for health. In particular, developers thought that the users might have been initially “externally motivated” to adopt Caren and have kept using it because they found it helpful. In fact, numerous telehealth solutions gained popularity during the COVID-19 pandemic because of their perceived usefulness [28,29]. However, the feasibility and application of telehealth in resource-limited settings and low- and middle-income countries are still debatable [30].

As indicated by our focus group participants, the Caren platform has seen a rise in platform users during COVID-19. However, the 2022 survey had a lower response rate than 2019, possibly due to being open for a shorter time. The quantitative results of our study showed that throughout the COVID-19 pandemic there was an increase of ICs and CRs who used the Caren platform several times per day. This seems understandable as due to COVID-19, face-to-face interaction was possible only to a limited extent. For both groups (ICs and CRs) we found an increase in the use of a shared digital agenda to make and view appointments (calendar), a messaging function to receive updates and communicate with formal and informal caregivers (messages), and digital notes to store important information (notes), suggesting that these support functionalities gained relevance for users throughout the COVID-19 pandemic and should therefore stay core platform elements. It stood out that the dossier functionality use (a connection to the electronic client record of the involved home care provider) decreased among ICs between 2019 and 2022. One would expect that being able to read along in the electronic client record of their CR would become more important for ICs in times where face-to-face contact is limited, especially for ICs who care on a distance of whom there were significantly more in 2022 as compared with 2019. However, the 2022 sample was significantly older than the 2019 sample, which might have hindered some ICs to use this more complex functionality (requiring additional steps such as 2-factor web-based authentication to access the records). Another explanation might stem from previous research which found that, before COVID-19, ICs who used Caren often criticized incomplete or delayed information in the electronic client record [31]. Due to the burden caused by COVID-19, care staff might have found it even more difficult to adequately fill in the electronic client record, reducing its usefulness for ICs.

### Strengths and Limitations

We believe that using an implementation framework such as the CFIR, which has frequently been used to retrospectively evaluate implementation or to prospectively design implementation strategies [32,33], provided a valuable tool for interpreting the results. Furthermore, to the best of our knowledge, this is the first study that compares the usage behavior and support functionality use of a well-established digital care tool before and after the COVID-19 pandemic. In addition, the comparison was made by using preexisting (as opposed to retrospective) data from before the pandemic.

The study also has some limitations that should be taken into consideration. First, the study was conducted within the context of the Dutch health care system, which may restrict its generalizability to other health care contexts. Second, the lack of input from significant stakeholders, such as formal caregivers or employees within the business department of Caren, is another notable limitation. The inclusion of these relevant stakeholders, as well as similar evaluations of non-Dutch digital health care platforms, should be considered for future research. Third, although the survey conducted in 2019 and 2022 contained similar questions, recoding response options was required to enable a comparison of the results. Finally, ICs and CRs from 2019 and 2022 differed regarding several demographic variables which might limit their comparability. A follow-up analysis (eg, multivariate regression) could identify variables that might have driven some of the observed differences between 2019 and 2022 use patterns. Furthermore, conducting subgroup analyses could provide insights into whether usage patterns differ across specific groups, such as younger versus older ICs.

### Conclusions

Our study identified several lessons learned from the continued implementation of the informal care platform Caren throughout the COVID-19 pandemic, such as finding innovative solutions to engage the target audience, considering automated user support, taking into account the fluctuation of user groups, and addressing concerns about data transparency in health care. Furthermore, our study shows which platform support functionalities gained relevance throughout the COVID-19 pandemic among both ICs and CRs. The findings can be used to improve the design and implementation of current and future digital platforms to support informal care toward the “new digital normal.”

## Supplementary material

10.2196/54734 Multimedia Appendix 1 Focus group guide.

10.2196/54734 Multimedia Appendix 2 Web-based survey (2019).

10.2196/54734 Multimedia Appendix 3 Web-based survey (2022).

10.2196/54734 Multimedia Appendix 4 Overview of lessons learned.

## Abbreviations

CFIR: Consolidated Framework for Implementation Research
CR: care recipient
IC: informal caregiver
