# Supplementary material for: Continued Implementation and Use of a Digital Informal Care Support Platform Before and After COVID-19: Multimethod Study
Source: JMIR Form Res. 2024 Dec 31;8:e54734. doi: 10.2196/54734 (PMC11706444; doi:10.2196/54734)
Supplement: Multimedia Appendix 1 [file formative-v8-e54734-s001.docx]

Focus group guide

**GENERAL INTRODUCTION (ca. 15 minutes)**

| Time | Topic | Questions/ instructions |
| --- | --- | --- |
| 5m | Welcome and introduction of ourselves | We would like to welcome you all to this meeting and thank you for your willingness to participate in this study.  Before we begin, we would like to introduce ourselves. |
| 5m | Background & goal of focus group | Explain background of the study:   - In collaboration with Nedap, a survey was conducted in 2019 (before Covid-19) and in 2022 (after Covid-19) via the Caren platform to gain more insight into the user group, the overall use of the platform and the usage of the different support functionalities of Caren, from the perspective of informal caregivers and care recipients. The results have been reported to Nedap previously. - The current study specifically focuses on a comparison between the 2019 and 2022 results in order to identify pre-post Covid-19 changes in use behavior of informal caregivers and care recipients. However, we would like to provide more context to these results including the technology developers’ perspective.   Explain goal of the focus group:  We have organized this meeting with you today to interpret the survey results from your perspective. Next to that, we would like to get a broader insight into question such as:   - What was the impact of Covid-19 on the platform Caren according to you? - How did you possibly react to the impacts Covid-19 may have caused? - Do you have any lessons learned regarding the continued implementation of Caren throughout the pandemic?   In our study, both quantitative results (surveys) and qualitative results (focus group) will be combined. We aim to translate findings into recommendations for implementation of digital care collaboration platforms to support informal care into a changing context (new digital normal). |
| 1m | General instructions | During this meeting we will discuss a number of topics with you step by step.  We would also like to emphasize a few things:   - There are **no right or wrong answers**. - If you want to add to someone else, if you agree or disagree with something, or if you want to give an example, you are completely free to do so, **discussions among yourselves are perfectly fine**. - We have a **tight schedule today**. This means that sometimes we may have to cut off an interesting discussion, for the sake of time. But of course we will do our best to give everyone a chance to say something. - In order not to miss any comments, we would like to **audio record** the session. However, in the final report no names will be included, so your comments will always remain **confidential**. |
| 5m | Signing informed consent and start recording | Questions so far?  In case of no questions: signing of informed consent forms and start recording |

**MAIN PART (ca. 70 minutes)**

| Time | Topic | Questions/ instructions |
| --- | --- | --- |
| 5m | Introduction round participants | Before we begin, we would like to ask you to introduce yourself shortly naming your:   - First name (to distinguish participants in the audio recording) - Age - A description of your role within Nedap/ Caren team - For long you have been working in your current role |
| 15m | Impact of Covid-19 | Have you observed any impacts on the platform Caren due to the Covid-19 circumstances (this can be both positive or negative)? Please take a moment to think about any impacts you may have observed related to the technology, inner setting (within NEDAP), outer setting (within Dutch healthcare system), users, or other impacts that come to your mind.  You can write aspects that come to your mind on the sticky notes in front of you.  After 5 minutes, we will discuss your answers (🡪 letting participants place their sticky notes into the corresponding CFIR category on the flip-over and ask for short explanation)  TECHNOLOGY  Impacts on certain (technical) characteristics of the platform or services surrounding the platform?  INNER SETTING (Organizational setting)  Impacts on those who develop and implement Caren (Caren team)?  OUTER SETTING (Healthcare setting)  Impacts on the broader implementation context (healthcare setting) in which Caren is embedded?  USERS  Impacts on the users of Caren? E.g. are there any specific needs that emerged due to Covid-19 from the users? |
| 15m | Concrete reactions | How did you react to these impacts due to Covid-19? Why?  Please take a moment to think about ways you possibly reacted to the impacts you may have observed related to the technology, inner setting, outer setting, users, or other reactions that come to your mind.  You can write aspects that come to your mind on the sticky notes in front of you.  After 5 minutes, we will discuss your answers (🡪 letting participants place their sticky notes into the corresponding CFIR category on the flip-over and ask for short explanation)  TECHNOLOGY  Changes in certain (technical) characteristics of the platform or services surrounding the platform?  INNER SETTING (Organizational setting)  Changes concerning those who develop and implement Caren (Caren team)? (New employees/new constellation of team? Different kind of expertise needed in the team due to Covid-19?)  OUTER SETTING (Healthcare setting)  Reactions to possible changes in the healthcare setting in which caren is embedded? (How did the Caren try to maintain its position within the healthcare system in a changing situation/ digitalization in fast pace?)  USERS  Reactions/ changes made in reaction to user needs? |
| 15m | Short presentation survey results  & reflection | Show 1 –2 slides presenting main findings  Starting with general question:  What is your first reaction about these main findings? Please explain.  (Let them brainstorm for 5 minutes).  After that (10 minutes):   - To what extent did you expect those results? - Do you have possible explanations for the results? |
| 15m | Lessons learned | - Do you have any lessons learned that you as developer/designers of Caren have gained throughout the pandemic? (Barriers and facilitators for continued implementation?) - How to maintain a fit between the technology (Caren), changing context, and users? (What kind of strategies did you apply (if any) to keep up with the changing situation? Changes in implementation strategy?) - Are there things that you as technology developers would have done differently looking back? If so, why? - In 4 domains: TECHNOLOGY, INNER SETTING, OUTER SETTING, USERS |

**CLOSING (ca. 5 minutes)**

| Time | Topic | Questions/ instructions |
| --- | --- | --- |
| 3m | Questions/ remarks? | We have arrived the end of this focus group session. Are there any aspects that we have not yet discussed in the previous sections that you would like to share? |
| 2m | Thank you | Thank you very much for your time and valuable help.  Your answers will be analyzed anonymously and will help us to provide context to the survey results as well as draw up some lessons learned regarding the continued implementation of platforms such as Caren during pandemics such as COVID-19.  Are you interested in the results of this research? If so, could we e-mail them to you? |
