# Supplementary material for: Continued Implementation and Use of a Digital Informal Care Support Platform Before and After COVID-19: Multimethod Study
Source: JMIR Form Res. 2024 Dec 31;8:e54734. doi: 10.2196/54734 (PMC11706444; doi:10.2196/54734)
Supplement: Multimedia Appendix 2 [file formative-v8-e54734-s002.pdf]

Vragenlijst Carenzorgt

Start of Block: Inleiding

Q1
Beste gebruiker van Carenzorgt,
Hartelijk dank voor uw bereidheid om deel te nemen aan dit onderzoek naar de gebruikerservaringen met Carenzorgt. Dit is een onderzoek van de Universiteit Twente in Enschede in samenwerking met Nedap Healthcare. Deelnemen aan het onderzoek kost ongeveer 5 minuten. Gegevens worden volledig anoniem en op veilige wijze verwerkt. Deelname aan het onderzoek is vrijwillig, wat betekent dat u op elk gewenst moment kunt stoppen met het invullen van de vragenlijst. Indien u vragen heeft over dit onderzoek of graag meer informatie wilt ontvangen, kunt u contact opnemen met Sophie van Oudheusden, email: a.s.vanoudheusden@student.utwente.nl.
Dit onderzoek wordt uitgevoerd onder leiding van Dr. Annemarie Braakman-Jansen, email: l.m.a.braakman-jansen@utwente.nl

Door deze vragenlijst te starten geeft u aan dat u:
- Bovenstaande informatie gelezen hebt en hiermee akkoord bent
- Vrijwillig meedoet aan het onderzoek
- 18 jaar of ouder bent

End of Block: Inleiding

Start of Block: Achtergrondinfromatie

Q1 Wat is uw leeftijd?

____

Q2 Geslacht

○ Man (1)

○ Vrouw (2)

○ Anders (3)

Q3 Ik gebruik Carenzorgt als

- ◯ Mantelzorger (1)
- ◯ Cliënt/Patiënt (2)

Q4 De reden van zorg is (meerdere antwoorden mogelijk)

- ▢ Dementie (1)
- ▢ Hart- en vaatziekten (2)
- ▢ Diabetes (3)
- ▢ COPD (4)
- ▢ Ouderdom (5)
- ▢ Anders (6)

Display This Question:

If Q3 = Mantelzorger

Q5 Welke woonsituatie is op u van toepassing?

- ◯ Ik woon in hetzelfde huis als degene voor wie ik zorg (1)
- ◯ Degene voor wie ik zorg woont niet bij mij in huis (2)

Display This Question:

If Q3 = Mantelzorger

Q6 Hoeveel uren per week verleent u ongeveer mantelzorg?

- ◯ Minder dan 8 uur per week (1)
- ◯ 8 tot 24 uur per week (2)
- ◯ 24 tot 40 uur per week (3)
- ◯ Meer dan 40 uur per week (4)

Display This Question:

If Q3 = Mantelzorger

Q7 In welke mate ervaart u belasting van uw mantelzorg taken?

- ◯ Geen tot zeer kleine mate (1)
- ◯ In kleine mate (2)
- ◯ In gemiddelde mate (3)
- ◯ In grote mate (4)
- ◯ In zeer grote mate (5)

Q8 Hoe vaak gebruikt u Carenzorgt gemiddeld?

- ◯ Maandelijks (1)
- ◯ Minder dan een keer per week (2)
- ◯ 1 tot 3 keer per week (3)
- ◯ 4 tot 6 keer per week (4)
- ◯ Elke dag (5)
- ◯ Meerdere keren per dag (6)

End of Block: Achtergrondinfromatie

Start of Block: Onderdelen Carenzorgt

Q9 Onderdelen Carenzorgt

	Welke onderdelen van Carenzorgt hebt u binnen de afgelopen maand gebruikt?	Wat is uw mening over deze onderdelen?				
	. (1)	Zeer ontevreden (1)	Ontevreden (2)	Neutraal (3)	Tevreden (4)	Zeer tevreden (5)
Kalender (25)	▢	◯	◯	◯	◯	◯
Berichten (26)	▢	◯	◯	◯	◯	◯
Notities (27)	▢	◯	◯	◯	◯	◯
Dossier (28)	▢	◯	◯	◯	◯	◯

Q30 Klik om de vraagtekst te schrijven

	Welke stellingen zijn op u van toepassing?	Wat is uw mening over deze functionaliteiten?				
	. (1)	Zeer ontevreden (1)	Ontevreden (2)	Neutraal (3)	Tevreden (4)	Zeer tevreden (5)

Ik ben via Carenzorgt verbonden met ten minste één andere mantelzorger (1)	▢	○	○	○	○	○
Ik heb Carenzorgt gekoppeld met een zorgaanbieder (2)	▢	○	○	○	○	○

End of Block: Onderdelen Carenzorgt

Start of Block: Blok 3

Q10 Geef aan in hoeverre u het eens bent met de volgende stellingen:

Q11 Carenzorgt is gemakkelijk te gebruiken

○ Helemaal mee eens (1)

○ Mee eens (2)

○ Noch eens noch oneens (3)

○ Niet mee eens (4)

○ Helemaal niet mee eens (5)

○ Niet van toepassing (6)

Q12 Carenzorgt is op een logische manier opgebouwd

- Helemaal mee eens (1)
- Mee eens (2)
- Noch eens noch oneens (3)
- Niet mee eens (4)
- Helemaal niet mee eens (5)
- Niet van toepassing (6)

Q13 Met Carenzorgt is de organisatie van zorgtaken is makkelijker

- Helemaal mee eens (1)
- Mee eens (2)
- Noch eens noch oneens (3)
- Niet mee eens (4)
- Helemaal niet mee eens (5)
- Niet van toepassing (6)

Display This Question:

If Q3 = Cliënt/Patiënt

Q14c Met Carenzorgt is het voor mij makkelijker om contact met mantelzorgers te onderhouden omtrent mijn zorg

- ◯ Helemaal mee eens (1)
- ◯ Mee eens (2)
- ◯ Noch eens noch oneens (3)
- ◯ Niet mee eens (4)
- ◯ Helemaal niet mee eens (5)
- ◯ Niet van toepassing (6)

Display This Question:

If Q3 = Mantelzorger

Q14m Met Carenzorgt is het voor mij makkelijker om contact met andere mantelzorgers te onderhouden omtrent de zorg voor mijn dierbare

- ◯ Helemaal mee eens (1)
- ◯ Mee eens (2)
- ◯ Noch eens noch oneens (3)
- ◯ Niet mee eens (4)
- ◯ Helemaal niet mee eens (5)
- ◯ Niet van toepassing (6)

Display This Question:

If Q3 = Cliënt/Patiënt

Q15c Met Carenzorgt is het voor mij makkelijker om contact met professionele zorgverleners te onderhouden omtrent mijn zorg

- ◯ Helemaal mee eens (1)
- ◯ Mee eens (2)
- ◯ Noch eens noch oneens (3)
- ◯ Niet mee eens (4)
- ◯ Helemaal niet mee eens (5)
- ◯ Niet van toepassing (6)

Display This Question:

If Q3 = Mantelzorger

Q15m Met Carenzorgt is het voor mij makkelijker om contact met professionele zorgverleners te onderhouden omtrent de zorg voor mijn dierbare

- ◯ Helemaal mee eens (1)
- ◯ Mee eens (2)
- ◯ Noch eens noch oneens (3)
- ◯ Niet mee eens (4)
- ◯ Helemaal niet mee eens (5)
- ◯ Niet van toepassing (6)

Q16 Carenzorgt zorgt voor een beter overzicht in het zorgproces

- Helemaal mee eens (1)
- Mee eens (2)
- Noch eens noch oneens (3)
- Niet mee eens (4)
- Helemaal niet mee eens (5)
- Niet van toepassing (6)

Display This Question:

If Q3 = Cliënt/Patiënt

Q17c Door het gebruik van Carenzorgt is het makkelijk om op de hoogte te blijven van mijn eigen situatie

- Helemaal mee eens (1)
- Mee eens (2)
- Noch eens noch oneens (3)
- Niet mee eens (4)
- Helemaal niet mee eens (5)
- Niet van toepassing (6)

Display This Question:

If Q3 = Mantelzorger

Q17m Door het gebruik van Carenzorgt is het makkelijk om op de hoogte te blijven van de situatie van mijn dierbare

- ○ Helemaal mee eens (1)
- ○ Mee eens (2)
- ○ Noch eens noch oneens (3)
- ○ Niet mee eens (4)
- ○ Helemaal niet mee eens (5)
- ○ Niet van toepassing (6)

Q18 Ik heb er vertrouwen in dat Carenzorgt zorgvuldig met onze gegevens omgaat

- ○ Helemaal mee eens (1)
- ○ Mee eens (2)
- ○ Noch eens noch oneens (3)
- ○ Niet mee eens (4)
- ○ Helemaal niet mee eens (5)
- ○ Niet van toepassing (6)

Q19 Ik zou Carenzorgt in de toekomst willen blijven gebruiken

- Helemaal mee eens (1)
- Mee eens (2)
- Noch eens noch oneens (3)
- Niet mee eens (4)
- Helemaal niet mee eens (5)
- Niet van toepassing (6)

Q20 Laatste vraag: Heeft u nog toevoegingen?

____

____

____

____

____

Display This Question:

If Q3 = Mantelzorger

And Q4 = Dementie

Q31 Wij vinden het erg belangrijk om mantelzorgers te kunnen betrekken bij het ontwikkelen en verbeteren van technologie die ondersteuning kan bieden. Zouden wij u mogen benaderen voor toekomstig onderzoek? Zo ja, dan kunt u hieronder uw e-mailadres voor ons achterlaten (vrijwillig). Vergeet niet om naar de volgende scherm te gaan om de vragenlijst af te sluiten.

____

End of Block: Blok 3
