# Supplementary material for: Continued Implementation and Use of a Digital Informal Care Support Platform Before and After COVID-19: Multimethod Study
Source: JMIR Form Res. 2024 Dec 31;8:e54734. doi: 10.2196/54734 (PMC11706444; doi:10.2196/54734)
Supplement: Multimedia Appendix 3 [file formative-v8-e54734-s003.pdf]

Vragenlijst

Beste gebruiker van Caren,

Hartelijk dank voor uw bereidheid om deel te nemen aan dit onderzoek. Dit onderzoek bestaat uit twee delen. In het eerste deel staan de gebruikerservaringen met Caren centraal. In het tweede deel laten we u kennis maken met een nieuw soort, nog te ontwikkelen, sensorsysteem. Het hier aan gekoppelde communicatieplatform moet nog vormgegeven worden en we willen graag weten hoe u hier over denkt.

Dit is een onderzoek van de Universiteit Twente in Enschede in samenwerking met de ontwikkelaar van Caren, Nedap Healthcare. Deelnemen aan het onderzoek kost ongeveer 15 minuten. Gegevens worden volledig anoniem en op veilige wijze verwerkt en zijn niet te herleiden tot uw gebruik van Caren. Deelname aan het onderzoek is vrijwillig, wat betekent dat u op elk gewenst moment kunt stoppen met het invullen van de vragenlijst. Uw antwoorden op de vragen worden maximaal 10 jaar bewaard door de Universiteit Twente, de samenvatting van de antwoorden wordt minimaal 10 jaar bewaard door Nedap.

Indien u vragen heeft over dit onderzoek of graag meer informatie wilt ontvangen, kunt u contact opnemen met Dineke Brouwer, email: n.e.j.brouwer@student.utwente.nl, Nikita Sharma, email n.sharma@uwente.nl of Dr. Annemarie Braakman-Jansen, email: l.m.a.braakman-jansen@utwente.nl.

* Required

1. Door deze vragenlijst te starten geeft u aan dat u *

Check all that apply.

- [ ] Bovenstaande informatie gelezen hebt en hiermee akkoord bent
- [ ] Vrijwillig meedoet aan het onderzoek
- [ ] 18 jaar of ouder bent

2. Wat is uw leeftijd? Graag antwoorden met cijfers *

____

3. Wat is uw geslacht? *

Mark only one oval.

- Man
- Vrouw
- Anders

4. Hoe vaak gebruikt u Caren? *

Mark only one oval.

- Een aantal keren per dag
- Een keer per dag
- 4-6 keer per week
- 1-3 keer per week
- Minder dan een keer per week
- Maandelijks

5. Hoe vaak gebruikt u de verschillende onderdelen van Caren? *

Mark only one oval per row.

	Nooit	Weinig	Soms	Vaak	Altijd
Kalender					
Berichten					
Dossier					
Notities					

6. Waarom gebruikt u Caren? *

Mark only one oval.

- Ik ontvang hulp *Skip to question 7*
- Ik geef hulp als mantelzorger (bijvoorbeeld familie, vrienden of buren) *Skip to question 37*
- Ik ben zorgprofessional (bijvoorbeeld verpleegkundige, thuiszorg of therapeut) *Skip to question 93*

Ik ontvang zorg

7. Wat is op u van toepassing? *

Mark only one oval.

- Mijn mantelzorgers (bijvoorbeeld familie, vrienden of buren) kunnen mijn zorgpagina zien *Skip to question 8*
- Mijn zorgverleners (bijvoorbeeld verpleegkundige, thuiszorg of therapeut) kunnen in Caren mijn zorgpagina zien *Skip to question 9*
- Mijn mantelzorgers en zorgverleners kunnen mijn zorgpagina zien *Skip to question 10*
- Ik weet het niet *Skip to question 12*
- Geen van bovenstaande *Skip to question 12*

Skip to question 12

Toepassing: Mijn mantelzorgers

8. Hoeveel mantelzorgers (bijvoorbeeld familie, vrienden of buren) van u gebruiken Caren? *

Mark only one oval.

- 1
- 2
- 3
- Meer dan 3

Skip to question 12

Toepassing: Mijn zorgverleners

9. Welke zorgverleners (bijvoorbeeld verpleegkundige, thuiszorg of therapeut) van u gebruiken Caren? Kies alle opties die op u van toepassing zijn *

Check all that apply.

- [ ] Behandelaar (zoals ergotherapeut, fysiotherapeut, maatschappelijk werker, arts, psycholoog)
- [ ] (wijk)verpleegkundige/zuster
- [ ] Verzorgende
- [ ] Begeleider
- [ ] Niet van toepassing

Skip to question 12

Toepassing: Mantelzorgers & zorgverleners

10. Hoeveel mantelzorgers (bijvoorbeeld familie, vrienden of buren) van u gebruiken Caren? *

Mark only one oval.

- ( ) 1
- ( ) 2
- ( ) 3
- ( ) Meer dan 3

11. Welke zorgverleners (bijvoorbeeld verpleegkundige, thuiszorg of therapeut) van u gebruiken Caren? Kies alle opties die op u van toepassing zijn *

Check all that apply.

- [ ] Behandelaar (zoals ergotherapeut, fysiotherapeut, maatschappelijk werker, arts, psycholoog)
- [ ] (wijk)verpleegkundige/zuster
- [ ] Verzorgende
- [ ] Begeleider
- [ ] Niet van toepassing

Skip to question 12

Vervolg: Ik ontvang zorg

12. Hoe lang gebruikt u Caren? *

Mark only one oval.

- ( ) Minder dan 1 jaar
- ( ) Tussen de 1 en 3 jaar
- ( ) Meer dan 3 jaar

13. Wat is de reden dat u zorg ontvangt? U kunt meerdere opties kiezen *

Check all that apply.

- [ ] Ouderdom
- [ ] Dementie of milde cognitieve beperking
- [ ] Lichamelijke beperking
- [ ] Psychische stoornis
- [ ] Slechtziend of slechthorend
- [ ] Verstandelijke handicap

Other: [ ] ______________________

14. 13. Hoelang ontvangt u mantelzorg (van bijvoorbeeld familie, vrienden of buren)? *

Mark only one oval.

- ( ) 0 tot 6 maanden
- ( ) 6 maanden tot 1 jaar
- ( ) 1 tot 2 jaar
- ( ) 2 tot 5 jaar
- ( ) Meer dan 5 jaar

15. Woont u alleen? *

Mark only one oval.

- Ja
- Nee

16. Wat is uw woonsituatie? *

Mark only one oval.

- Ik woon in een eigen (huur)woning
- Ik woon in een aanleunwoning/ouderenwoning
- Ik woon in een verzorgingshuis/verpleeghuis

17. Hoe vaak wordt u bezocht door iemand die zorg aan u verleent? *

Mark only one oval.

- Een aantal keren per dag
- Een keer per dag
- 4-6 keer per week
- 1-3 keer per week
- Minder dan een keer per week

Uitleg over het slimme kastje

De universiteit doet onderzoek naar een nieuwe onopvallende detectietechnologie om bepaalde risico's te signaleren. Dit houdt in dat u niks hoeft te dragen op uw lichaam en geen apparaat hoeft te gebruiken. Zoals u kunt zien in de afbeelding kan er een klein kastje, denk aan een lichtsensor, op een onopvallende plek van uw woning geplaatst worden. Dit slimme kastje kan verschillende activiteiten volgen en belangrijke veranderingen waarnemen, zoals minder drinken of eten, verandering in de hartslag of ademhaling, (on)rustiger slapen, maar ook situaties zoals een val. Houd dit slimme kastje in gedachten bij het beantwoorden van de volgende vragen:

Slimme kastje in huis

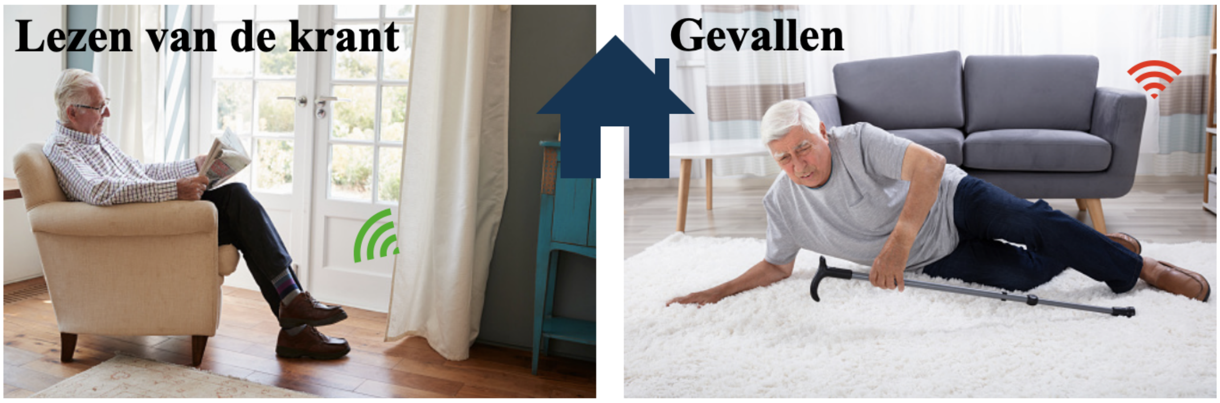

18. Zou u dit slimme kastje in uw woning accepteren om uw dagelijkse activiteiten zoals eten, drinken en het innemen van medicijnen te volgen? *

Mark only one oval.

- Helemaal niet
- Niet
- Misschien
- Wel
- Zeker wel

19. Zou u dit slimme kastje in uw woning accepteren om noodsituaties zoals een val te monitoren? *

Mark only one oval.

- Helemaal niet
- Niet
- Misschien
- Wel
- Zeker wel

20. Denkt u dat dit slimme kastje uw zorgverleners kan ondersteunen bij het leveren van betere zorg en zorg op het moment dat die gewenst is? *

Mark only one oval.

- Helemaal niet
- Niet
- Misschien
- Wel
- Zeker wel

21. Denkt u dat dit slimme kastje u zal helpen om langer zelfstandig en veilig thuis te kunnen wonen? *

Mark only one oval.

- Helemaal niet
- Niet
- Misschien
- Wel
- Zeker wel

22. Mogen uw zorgverleners en mantelzorgers ook de informatie uit het slimme kastje ontvangen? *

Mark only one oval.

- Ja, alleen mijn zorgverleners (bijvoorbeeld verpleegkundige, thuiszorg of therapeut) *Skip to question 25*
- Ja, alleen mijn mantelzorgers (bijvoorbeeld familie, vrienden of buren) *Skip to question 28*
- Ja, zowel mijn mantelzorgers als mijn zorgverleners *Skip to question 31*
- Dat weet ik niet zeker

23. Wat is uw hoogst behaalde diploma? (optioneel)

Mark only one oval.

- Geen diploma
- Basisonderwijs
- VMBO, MBO 1-2
- HAVO, VWO, HBS, MBO 3-4
- Bachelor (HBO/WO)
- Master (HBO/WO)
- Other: ____________

24. Wilt u deelnemen aan een volgend onderzoek over het verlenen van zorg? *

Mark only one oval.

- Ja *Skip to question 35*
- Nee
- Misschien *Skip to question 35*

Informatie delen: mijn zorgverleners

25. Welke informatie mogen uw zorgverleners (bijvoorbeeld verpleegkundige, thuiszorg of therapeut) zien? *

Mark only one oval.

- Alle informatie
- Alleen informatie over noodsituaties
- Alleen informatie over dagelijks welzijn

26. Wat is uw hoogst behaalde diploma? (optioneel)

Mark only one oval.

- Geen diploma
- Basisonderwijs
- VMBO, MBO 1-2
- HAVO, VWO, HBS, MBO 3-4
- Bachelor (HBO/WO)
- Master (HBO/WO)
- Other: ______________________

27. Wilt u deelnemen aan een volgend onderzoek over het verlenen van zorg? *

Mark only one oval.

- Ja *Skip to question 35*
- Nee
- Misschien *Skip to question 35*

Skip to question 35

Informatie delen: mijn mantelzorgers

28. Welke informatie mogen uw mantelzorgers (bijvoorbeeld familie, vrienden of buren) zien? *

Mark only one oval.

- Alle informatie
- Alleen informatie over noodsituaties
- Alleen informatie over dagelijks welzijn

29. Wat is uw hoogst behaalde diploma? (optioneel)

Mark only one oval.

- Geen diploma
- Basisonderwijs
- VMBO, MBO 1-2
- HAVO, VWO, HBS, MBO 3-4
- Bachelor (HBO/WO)
- Master (HBO/WO)
- Other: ______________________

30. Wilt u deelnemen aan een volgend onderzoek over het verlenen van zorg? *

Mark only one oval.

- Ja *Skip to question 35*
- Nee
- Misschien *Skip to question 35*

Skip to question 35

Informatie delen: Zorgverleners & mantelzorgers

31. Welke informatie mogen uw zorgverleners (bijvoorbeeld verpleegkundige, thuiszorg of therapeut) zien? *

Mark only one oval.

- Alle informatie
- Alleen informatie over noodsituaties
- Alleen informatie over dagelijks welzijn

32. Welke informatie mogen uw mantelzorgers (bijvoorbeeld familie, vrienden of buren) zien? *

Mark only one oval.

- Alle informatie
- Alleen informatie over noodsituaties
- Alleen informatie over dagelijks welzijn

33. Wat is uw hoogst behaalde diploma? (optioneel)

Mark only one oval.

- Geen diploma
- Basisonderwijs
- VMBO, MBO 1-2
- HAVO, VWO, HBS, MBO 3-4
- Bachelor (HBO/WO)
- Master (HBO/WO)
- Other: ______________________

34. Wilt u deelnemen aan een volgend onderzoek over het verlenen van zorg? *

Mark only one oval.

- Ja *Skip to question 35*
- Nee
- Misschien *Skip to question 35*

Skip to question 35

Contactgegevens

Vul dan hier alstublieft uw mailadres in, of als wij u mogen bellen uw telefoonnummer, zodat wij contact met u kunnen opnemen.

35. Vul hier uw mailadres in

____

36. Vul hier uw telefoonnummer in

____

Ik ben een mantelzorger

37. Aan hoeveel mensen verleent u mantelzorg? Graag antwoorden met cijfers *

____

38. Hoe lang bent u al actief als mantelzorger? *

Mark only one oval.

- Minder dan 1 jaar
- Tussen de 1 en 3 jaar
- Meer dan 3 jaar

39. Hoe lang gebruikt u Caren? *

Mark only one oval.

- Minder dan 1 jaar
- Tussen de 1 en 3 jaar
- Meer dan 3 jaar

40. Bent u tevreden over Caren? *

Mark only one oval.

- Zeer tevreden
- Tevreden
- Neutraal
- Ontevreden
- Zeer ontevreden

Denk bij het beantwoorden van de volgende vragen aan één specifieke persoon aan wie u mantelzorg verleent

41. Wat is de leeftijd van de persoon aan wie u zorg verleent? Graag antwoorden met cijfers *

____

42. Aan hoeveel zorgorganisaties bent u gekoppeld in Caren? *

Mark only one oval.

- 1
- 2
- 3
- Meer dan 3

43. Welke zorgverleners zijn aan u gekoppeld in Caren? Kies alle opties die op u van toepassing zijn *

Check all that apply.

- [ ] Behandelaar (zoals ergotherapeut, fysiotherapeut, maatschappelijk werker, arts psycholoog)
- [ ] (wijk)verpleegkundige/zuster
- [ ] Verzorgende
- [ ] Begeleider
- [ ] Niet van toepassing

44. Hoe vaak bezoekt u de persoon voor wie u zorgt? *

Mark only one oval.

- Een aantal keren per dag
- Een keer per dag
- 4-6 keer per week
- 1-3 keer per week
- Minder dan een keer per week
- Maandelijks

45. Wat is de reden dat u zorg verleent? U kunt meerdere opties kiezen *

Check all that apply.

- [ ] Ouderdom
- [ ] Dementie of milde cognitieve beperking
- [ ] Lichamelijke beperking
- [ ] Psychische stoornis
- [ ] Slechtziend of slechthorend
- [ ] Verstandelijke handicap

Other: [ ] ______________________

46. Hoe lang verleent u al zorg aan deze persoon? *

Mark only one oval.

- 0 tot 6 maanden
- 6 maanden tot 1 jaar
- 1 tot 2 jaar
- 2 tot 5 jaar
- Meer dan 5 jaar

47. Wat is uw relatie tot de persoon voor wie u zorgt? Ik ben *

Mark only one oval.

- Echtgenoot / partner
- Dochter/zoon
- Schoondochter / schoonzoon
- Kleindochter / kleinzoon
- buren / vriend(in)
- Other: ____________________

48. Welke woonsituatie is op u van toepassing? *

Mark only one oval.

- Ik woon in hetzelfde huis als de persoon voor wie ik zorg *Skip to question 50*
- Degene voor wie ik zorg woont niet bij mij in huis *Skip to question 49*

Woont niet bij mij in huis

49. Hoe ver vandaan woont u van de persoon voor wie u zorgt? *

Mark only one oval.

- Ik woon erg dichtbij (op minder dan 5 minuten reistijd)
- Ik woon op ongeveer 15 minuten reisafstand
- Ik woon op ongeveer 30 minuten reisafstand
- Ik woon op ongeveer 1 uur reisafstand
- Ik woon op meer dan een uur reistijd

Skip to question 50

Vragen over het sensorsysteem

De universiteit doet onderzoek naar een nieuwe onopvallende detectietechnologie om bepaalde risico's te signaleren. Dit houdt in dat u niks hoeft te dragen op uw lichaam en geen apparaat hoeft te gebruiken. Zoals u kunt zien in de afbeelding kan er een klein kastje, denk aan een lichtsensor, op een onopvallende plek van uw woning geplaatst worden. Dit slimme kastje kan verschillende activiteiten volgen en belangrijke veranderingen waarnemen, zoals minder drinken of eten, verandering in de hartslag of ademhaling, (on)rustiger slapen, maar ook situaties zoals een val. Houd dit slimme kastje in gedachten bij het beantwoorden van de volgende vragen:

Slimme kastje in huis

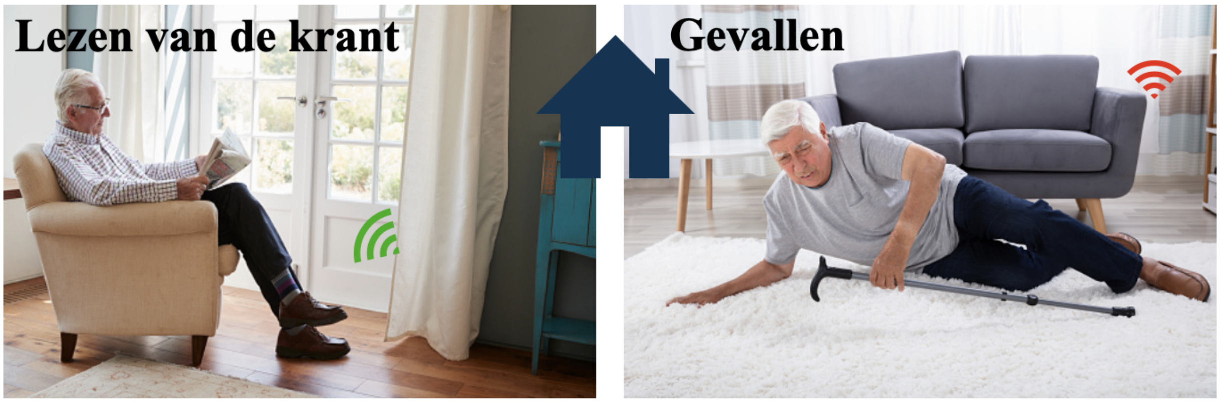

50. Zou u dit slimme kastje in de woning van uw naaste accepteren om hun dagelijkse activiteiten zoals eten, drinken en het innemen van medicijnen te monitoren? *

Mark only one oval.

- Helemaal niet
- Niet
- Misschien
- Wel
- Zeker wel

51. Zou u dit slimme kastje in de woning van uw naaste accepteren om noodsituaties zoals een val te monitoren? *

Mark only one oval.

- Helemaal niet
- Niet
- Misschien
- Wel
- Zeker wel

52. Denkt u dat dit slimme kastje u kan ondersteunen bij het leveren van betere zorg en zorg op het moment dat die gewenst is? *

Mark only one oval.

- Helemaal niet
- Niet
- Misschien
- Wel
- Zeker wel

53. Denkt u dat dit slimme kastje de persoon voor wie u zorgt zal helpen om langer zelfstandig en veilig thuis te kunnen wonen? *

Mark only one oval.

- Helemaal niet
- Niet
- Misschien
- Wel
- Zeker wel

54. Zou u zo’n slim kastje in het huis van de persoon voor wie u zorgt willen gebruiken? *

Mark only one oval.

- Zeker wel
- Waarschijnlijk wel
- Misschien
- Waarschijnlijk niet
- Zeker niet

Informatieplatform

Nadat het slimme kastje informatie verzameld heeft bij de persoon voor wie u zorgt, is het natuurlijk belangrijk dat deze informatie op de juiste manier bij u komt, via een platform. Deze informatie helpt u bij het plannen van bezoeken, het krijgen van zekerheid wanneer alles in orde is of geeft een waarschuwing in geval van nood. Graag willen we begrijpen welke informatie voor u belangrijk is.

Informatieplatform met slimme kastje

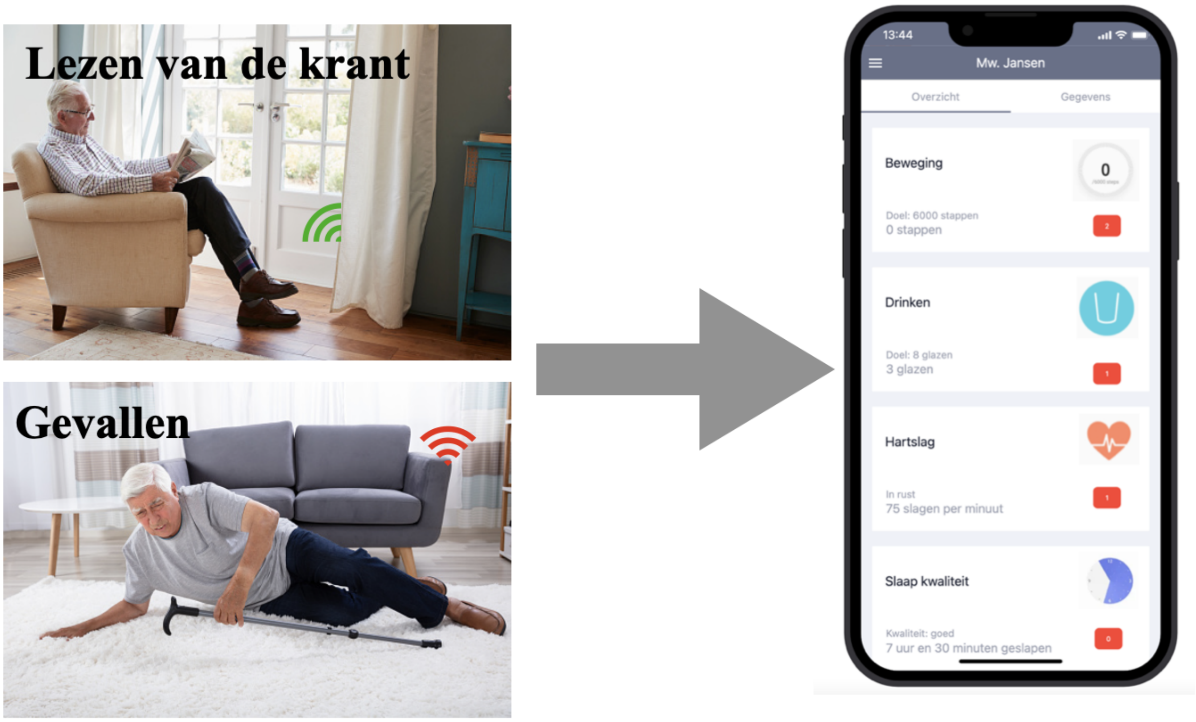

55. Welke informatie wilt u ontvangen? (meerdere antwoorden mogelijk) *

Check all that apply.

- [ ] Alle informatie die verzameld kan worden
- [ ] Alleen informatie over noodsituaties (zoals een val)
- [ ] Informatie over slaapkwaliteit/nachtelijke onrust
- [ ] Informatie over medicatie inname
- [ ] Informatie over inname van water en voedsel
- [ ] Informatie over persoonlijke hygiëne (zoals wassen, tandenpoetsen en aankleden)
- [ ] Informatie over onrust overdag/agitatie
- [ ] Ik wil deze informatie niet ontvangen

56. Op welk apparaat wilt u informatie ontvangen over het slimme kastje? *

Check all that apply.

- [ ] Laptop/computer
- [ ] Tablet
- [ ] Telefoon

Other: [ ] ______________________

57. Welke van onderstaande scenario's is het meest herkenbaar voor u? *

Mark only one oval.

- ( ) De persoon voor wie ik zorg valt (regelmatig) *Skip to question 58*
- ( ) De persoon voor wie ik zorg ervaart nachtelijke onrust *Skip to question 64*
- ( ) De persoon voor wie ik zorg ervaart agitatie (stress/geprikkeldheid) *Skip to question 71*
- ( ) Geen van bovenstaande scenario's is herkenbaar voor mij *Skip to question 77*

Een val

Het is maandagochtend, rond 11.00 uur heeft het slimme kastje een val gedetecteerd. U wordt geïnformeerd door het platform dat uw naaste gevallen is en op de badkamervloer ligt.

58. Wat vindt u van deze situatie? *

Mark only one oval.

- Een noodgeval
- Wel urgent, maar geen noodgeval
- Normale situatie

59. Op welke manier wilt u ingelicht worden door het slimme kastje in het geval dat uw naaste is gevallen? *

Check all that apply.

- [ ] Een notificatie of pop-up, zoals die van Caren
- [ ] Een tekstbericht/SMS
- [ ] Een (prioriteit) email
- [ ] Een telefoontje of voicemail
- [ ] Een update op het platform, zodat ik zelf kan kijken wanneer ik tijd heb

Other: [ ] ____________________

60. Moet het kastje u een melding sturen wanneer uw naaste gevallen is? *

Mark only one oval.

- Ja, meteen
- Ja, na een paar minuten als mijn naaste niet is opgestaan
- Ja, maar alleen op bepaalde momenten die ik zelf heb ingesteld
- Nee, ik controleer zelf zodra ik tijd heb

61. Wie moet er een bericht ontvangen wanneer uw naaste gevallen is? *

Mark only one oval.

- Alleen ik zelf moet een melding ontvangen
- Alleen alle mantelzorgers (bijvoorbeeld familie, vrienden of buren) moeten een melding ontvangen
- Alleen de zorgverleners (bijvoorbeeld verpleegkundige, thuiszorg of therapeut) moeten een melding ontvangen
- Alle mantelzorgers en zorgverleners moeten een melding ontvangen
- Alleen ikzelf en de zorgverleners (bijvoorbeeld verpleegkundige, thuiszorg of therapeut) moeten een melding ontvangen
- Alleen ikzelf en de mantelzorgers (bijvoorbeeld familie, vrienden of buren) moeten een melding ontvangen
- Niet toepasbaar

62. Wat moet het slimme kastje doen als u niet meteen (binnen een minuut) de melding opent als uw naaste is gevallen? *

Mark only one oval.

- Wacht 5 minuten, zodat ik de tijd heb om de melding te bekijken
- Neem contact op met een andere mantelzorger van mijn naaste
- Neem contact op met de zorgverleners van mijn naaste
- Bel de huisarts/112
- Other: ______________________

63. Als het slimme kastje mij informeert over de val, dan wil ik deze informatie het liefste als volgt ontvangen: *

Ruwe data: Het slimme kastje heeft een val in de badkamer geregistreerd en uw naaste is niet in staat om op te staan. De bloeddruk van uw naaste had een plotselinge bloeddrukdaling van 20 mm Hg.

Geïnterpreteerde data: Het slimme kastje heeft een val gedetecteerd, uw naaste is gevallen door een bloeddrukdaling.

Interpretatie en suggestie: Het slimme kastje heeft een ernstige val en bloeddrukdaling geobserveerd. U wordt geadviseerd om uw naaste te bezoeken of iemand anders te vragen om langs te gaan.

Mark only one oval.

- Ruwe data: de informatie die ontvangen is door het slimme kastje, zonder enige interpretatie. Zie de tekstballon hierboven!
- Geïnterpreteerde data: de informatie van het slimme kastje wordt direct geanalyseerd door het kastje, vervolgens wordt de informatie gedeeld door het platform. Zie de tekstballon hierboven!
- Interpretatie en suggestie: de informatie wordt direct geanalyseerd door het slimme kastje, samen met de informatie wordt er een suggestie gedaan voor welke actie u kunt ondernemen. Zie de tekstballon hierboven!
- Other: ______________________

Skip to question 77

Nachtelijke onrust

U heeft uw naaste om 1 uur 's nachts aan de telefoon gehad waarin hij/zij u vertelde dat hij/zij slecht kan slapen. Om 3 uur 's nachts observeert het slimme kastje dat uw naaste steeds aan het draaien is in bed, uit bed komt en door het huis dwaalt.

64. Wat vindt u van deze situatie? *

Mark only one oval.

- ( ) Een noodgeval
- ( ) Wel urgent, maar geen noodgeval
- ( ) Normale situatie

65. Op welke manier wilt u ingelicht worden door het slimme kastje in het geval dat uw naaste nachtelijke onrust vertoont? *

Check all that apply.

- [ ] Een notificatie of pop-up, zoals die van Caren
- [ ] Een tekstbericht/SMS
- [ ] Een (prioriteit) email
- [ ] Een telefoontje of voicemail
- [ ] Een update op het platform, zodat ik zelf kan kijken wanneer ik tijd heb

Other: [ ] ____________________

66. Wilt u een melding ontvangen elke keer als uw naaste opstaat uit bed? *

Mark only one oval.

- ( ) Ja
- ( ) Nee
- ( ) Ik wil zelf deze optie kunnen aanpassen

67. Wilt u een melding ontvangen elke keer als uw naast weer naar bed gaat? *

Mark only one oval.

- ( ) Ja
- ( ) Nee
- ( ) Ik wil zelf deze optie kunnen aanpassen

68. Wilt u graag een gedetailleerd rapport ontvangen over de nachtelijke onrust van uw naaste? *

Mark only one oval.

- Ja, iedere dag
- Ja, observeer een paar dagen en stuur een rapport als de onrust aanhoudt.
- Ja, observeer een paar weken en stuur een rapport wanneer de onrust aanhoudt.
- Nee, verstuur deze informatie naar de zorgverleners
- Other: ______________________

69. Wat moet het kastje doen als u niet meteen (binnen een minuut) de melding opent als uw naaste nachtelijke onrust vertoont? *

Mark only one oval.

- Wacht 5 minuten, zodat ik de tijd heb om de melding te bekijken
- Neem contact op met een andere mantelzorger van mijn naaste
- Neem contact op met de zorgverleners van mijn naaste
- Bel de huisarts/112
- Other: ______________________

70. Als het kastje mij informeert over de nachtelijke onrust, dan wil ik deze informatie het liefste als volgt ontvangen *

Ruwe data: Het slimme kastje heeft geconstateerd dat uw naaste tien keer is opgestaan vannacht. De afgelopen 5 uur was uw geliefde veel aan het draaien in bed.

Geïnterpreteerde data: Het slimme kastje heeft geconstateerd dat uw naaste last heeft van nachtelijke onrust, waardoor uw naaste niet goed kan slapen.

Interpretatie en suggestie: Het slimme kastje heeft geconstateerd dat uw naaste al een paar nachten last heeft van nachtelijke onrust. Wij raden u aan om uw naaste te bezoeken in de aankomende dagen of iemand anders te vragen om even langs te gaan.

Mark only one oval.

- Ruwe data: de informatie die ontvangen is door het slimme kastje, zonder enige interpretatie. Zie de tekstballon hierboven!
- Geïnterpreteerde data: de informatie van het slimme kastje wordt direct geanalyseerd door het kastje, vervolgens wordt de informatie gedeeld door het platform. Zie de tekstballon hierboven!
- Interpretatie en suggestie: de informatie wordt direct geanalyseerd door het slimme kastje, samen met de informatie wordt er een suggestie gedaan voor welke actie u kunt ondernemen. Zie de tekstballon hierboven!
- Other: ____________________

Skip to question 77

Agitatie (stress of geprikkeldheid overdag)

Het slimme kastje detecteert steeds vaker dat uw naaste overdag onrustig en geprikkeld is. Zo gooit hij/zij de krant op de grond, schopt hij/zij tegen de tafelpoot of reageert boos wanneer de bus stopt bij de halte voor het huis.

71. Wat vindt u van deze situatie? *

Mark only one oval.

- Een noodgeval
- Wel urgent, maar geen noodgeval
- Normale situatie

72. Op welke manier wilt u ingelicht worden door het slimme kastje in het geval dat uw naaste onrust of geprikkeldheid vertoont overdag? *

Check all that apply.

- Een notificatie of pop-up, zoals die van Caren
- Een tekstbericht/SMS
- Een (prioriteit) email
- Een telefoontje of voicemail
- Een update op het platform, zodat ik zelf kan kijken wanneer ik tijd heb

Other: ____________________

73. Wanneer wilt u deze informatie ontvangen:

Mark only one oval.

- Op elk moment van de dag
- Alleen op bepaalde momenten, die ik zelf aangegeven heb
- Nooit

74. Wilt u graag een gedetailleerd rapport ontvangen over de onrust of geprikkeldheid van uw naaste? Ja, iedere dag *

Mark only one oval.

- Ja, iedere dag
- Ja, observeer een paar dagen en stuur een rapport als de onrust aanhoudt.
- Ja, observeer een paar weken en stuur een rapport wanneer de onrust aanhoudt.
- Nee, verstuur deze informatie naar de zorgverleners
- Other: ____________________

75. Wat moet het kastje doen als u niet meteen (binnen een minuut) de melding opent als uw naaste onrust of geprikkeldheid vertoont? *

Mark only one oval.

- Wacht 5 minuten, zodat ik de tijd heb om de melding te bekijken
- Neem contact op met een andere mantelzorger van mijn naaste
- Neem contact op met de zorgverleners van mijn naaste
- Bel de huisarts/112
- Other: ____________________

76. Als het kastje mij informeert over stress of geprikkeldheid overdag, dan wil ik deze informatie het liefste als volgt ontvangen. *

Ruwe data: Het slimme kastje heeft geconstateerd dat uw naaste vaak tegen voorwerpen aanschopt en veel snelle bewegingen met de handen maakt

Geïnterpreteerde data: Het slimme kastje heeft geconstateerd dat uw naaste onrust vertoont sinds een paar uur.

Interpretatie en suggestie: Het slimme kastje heeft geconstateerd dat uw naaste onrust vertoont, waardoor hij/zij niet kan rusten. Wij adviseren u om uw naaste vandaag nog te bezoeken of te vragen of iemand anders even langs kan gaan.

Mark only one oval.

- Ruwe data: de informatie die ontvangen is door het slimme kastje, zonder enige interpretatie. Zie de tekstballon hierboven!
- Geïnterpreteerde data: de informatie van het slimme kastje wordt direct geanalyseerd door het kastje, vervolgens wordt de informatie gedeeld door het platform. Zie de tekstballon hierboven!
- Interpretatie en suggestie: de informatie wordt direct geanalyseerd door het slimme kastje, samen met de informatie wordt er een suggestie gedaan voor welke actie u kunt ondernemen. Zie de tekstballon hierboven!
- Other: ____________________

Skip to question 77

Normale dag

Het is een normale dag waarin het slimme kastje niks afwijkends heeft waargenomen. Uw naaste heeft een gezonde dag gehad waarin er voldoende gegeten, bewogen en geslapen is.

77. Op welke manier wilt u ingelicht worden door het slimme kastje in het geval dat uw naaste een normale dag heeft? *

Check all that apply.

- [ ] Een notificatie of pop-up, zoals die van Caren
- [ ] Een tekstbericht/SMS
- [ ] Een (prioriteit) email
- [ ] Een telefoontje of voicemail
- [ ] Een update op het platform, zodat ik zelf kan kijken wanneer ik tijd heb

Other: [ ] ______________________

78. Wanneer wilt u deze informatie ontvangen? *

Mark only one oval.

- ( ) Op elk moment van de dag
- ( ) Alleen op bepaalde momenten, die ik zelf aangegeven heb
- ( ) Nooit

79. Hoe vaak wilt u een update ontvangen? *

Mark only one oval.

- ( ) Ik wil een update ontvangen na elke activiteit (geslapen, gegeten, bewogen)
- ( ) Stuur mij de informatie aan het einde van de dag
- ( ) Ik hoef hier geen informatie over te ontvangen
- ( ) Ik zal zelf op het platform kijken zodra ik tijd heb
- ( ) Ik wil zelf graag in kunnen vullen welke informatie ik ontvang en wanneer
- ( ) Other: ______________________

80. Wat moet het slimme kastje doen als u niet meteen (binnen een minuut) de melding opent dat uw naaste een normale dag heeft? *

Mark only one oval.

- ( ) Wacht 5 minuten, zodat ik de tijd heb om de melding te bekijken
- ( ) Neem contact op met een andere mantelzorger van mijn naaste
- ( ) Neem contact op met de zorgverleners van mijn naaste
- ( ) Bel de huisarts/112
- ( ) Other: ______________________

81. Als het slimme kastje mij informeert over een normale dag, dan wil ik deze informatie het liefste als volgt ontvangen *

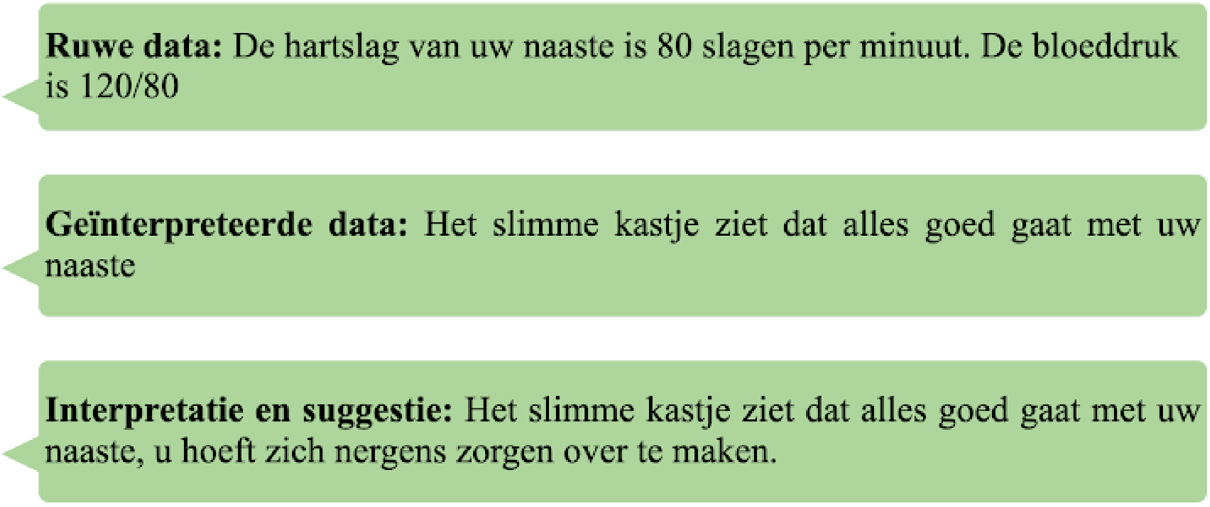

Mark only one oval.

- ( ) Ruwe data: de informatie die ontvangen is door het slimme kastje, zonder enige interpretatie. Zie de tekstballon hierboven!
- ( ) Geïnterpreteerde data: de informatie van het slimme kastje wordt direct geanalyseerd door het kastje, vervolgens wordt de informatie gedeeld door het platform. Zie de tekstballon hierboven!
- ( ) Interpretatie en suggestie: de informatie wordt direct geanalyseerd door het slimme kastje, samen met de informatie wordt er een suggestie gedaan voor welke actie u kunt ondernemen. Zie de tekstballon hierboven!
- ( ) Other: ______________________

Technologie acceptatie

Houd het eerder genoemde slimme kastje in combinatie met een communicatieplatform als product in gedachten bij het beantwoorden van de volgende vragen.
Geef aan in hoeverre u het eens bent met de volgende stellingen:

82. Het gebruik van dit product zou mij de zorgverlening makkelijker maken. *

Mark only one oval.

- Helemaal mee oneens
- Mee oneens
- Deels mee oneens
- Neutraal (niet mee oneens/ niet mee eens)
- Deels mee eens
- Mee eens
- Helemaal mee eens

83. Het gebruik van dit product zou mij in staat stellen de zorg te leveren die mijn naaste nodig heeft. *

Mark only one oval.

- Helemaal mee oneens
- Mee oneens
- Deels mee oneens
- Neutraal (niet mee oneens/ niet mee eens)
- Deels mee eens
- Mee eens
- Helemaal mee eens

84. Het gebruik van dit product zou mijn effectiviteit bij het zorgen voor mijn naaste vergroten. *

Mark only one oval.

- Helemaal mee oneens
- Mee oneens
- Deels mee oneens
- Neutraal (niet mee oneens/ niet mee eens)
- Deels mee eens
- Mee eens
- Helemaal mee eens

85. Ik zou dit product nuttig vinden in de zorg voor mijn naaste. *

Mark only one oval.

- Helemaal mee oneens
- Mee oneens
- Deels mee oneens
- Neutraal (niet mee oneens/ niet mee eens)
- Deels mee eens
- Mee eens
- Helemaal mee eens

86. Het leren omgaan met dit product zou voor mij gemakkelijk zijn. *

Mark only one oval.

- Helemaal mee oneens
- Mee oneens
- Deels mee oneens
- Neutraal (niet mee oneens/ niet mee eens)
- Deels mee eens
- Mee eens
- Helemaal mee eens

87. Ik zou dit product makkelijk vinden in gebruik. *

Mark only one oval.

- Helemaal mee oneens
- Mee oneens
- Deels mee oneens
- Neutraal (niet mee oneens/ niet mee eens)
- Deels mee eens
- Mee eens
- Helemaal mee eens

88. Wanneer ik toegang zou hebben tot dit product, zou ik het gaan gebruiken bij de zorg voor mijn naaste. *

Mark only one oval.

- Helemaal mee oneens
- Mee oneens
- Deels mee oneens
- Neutraal (niet mee oneens/ niet mee eens)
- Deels mee eens
- Mee eens
- Helemaal mee eens

89. Wat is uw hoogst behaalde diploma? (optioneel)

Mark only one oval.

- Geen diploma
- Basisonderwijs
- VMBO, MBO 1-2
- HAVO, VWO, HBS, MBO 3-4
- Bachelor (HBO/WO)
- Master (HBO/WO)
- Other: ____________________

90. Wilt u deelnemen aan een volgend onderzoek over het verlenen van zorg? *

Mark only one oval.

- Ja *Skip to question 91*
- Nee
- Misschien *Skip to question 91*

Contactgegevens - Mantelzorger

Vul dan hier alstublieft uw mailadres in, of als wij u mogen bellen uw telefoonnummer, zodat wij contact met u kunnen opnemen.

91. Vul hier uw mailadres in

____

92. Vul hier uw telefoonnummer in

____

Vragen voor zorgverleners

93. Aan hoeveel cliënten bent u gekoppeld in Caren? *

Mark only one oval.

- 1
- 2
- 3
- Meer dan 3

94. Hoe lang gebruikt u Caren? *

Mark only one oval.

- Minder dan 1 jaar
- Tussen de 1 en 3 jaar
- Meer dan 3 jaar

95. Heeft u toegang tot het elektronisch cliënten dossier van de cliënt(en) (bijvoorbeeld via ONS, HiX of Ysis)? *

Mark only one oval.

- Ja
- Ja, maar niet voor alle gekoppelde clienten
- Nee

96. Wat is/zijn de belangrijkste reden(en) om Caren als zorgprofessional te gebruiken (meerdere opties mogelijk) *

Check all that apply.

- [ ] Communicatie met de andere mensen in het mantelzorgnetwerk
- [ ] Meelezen in het zorgdossier
- [ ] Agenda afspraken inzien en beheren

Other: [ ] ______________________

97. Wat is uw hoogst behaalde diploma? (optioneel)

Mark only one oval.

- Geen diploma
- Basisonderwijs
- VMBO, MBO 1-2
- HAVO, VWO, HBS, MBO 3-4
- Bachelor (HBO/WO)
- Master (HBO/WO)
- Other: __________

98. Wilt u deelnemen aan een volgend onderzoek over het verlenen van zorg? *

Mark only one oval.

- Ja *Skip to question 99*
- Nee
- Misschien *Skip to question 99*

Contactgegevens - Zorgprofessional

Vul dan hier alstublieft uw mailadres in, of als wij u mogen bellen uw telefoonnummer, zodat wij contact met u kunnen opnemen.

99. Vul hier uw mailadres in

____

100. Vul hier uw telefoonnummer in

____

This content is neither created nor endorsed by Google.

Google Forms
