# Supplementary material for: Continued Implementation and Use of a Digital Informal Care Support Platform Before and After COVID-19: Multimethod Study
Source: JMIR Form Res. 2024 Dec 31;8:e54734. doi: 10.2196/54734 (PMC11706444; doi:10.2196/54734)
Supplement: Multimedia Appendix 4 [file formative-v8-e54734-s004.docx]

**Appendix 4:** Overview of lessons learned from the continued implementation of informal care support platform Caren throughout the COVID-19 pandemic

| **CFIR Domain** | **Recommendations** | **Explanation** |
| --- | --- | --- |
| Inner setting | Act promptly to maintain workflow. | Amidst the COVID-19 pandemic, Caren's team experienced escalated pressure in addressing user support. Although the type of help requests from users did not deviate significantly from those received before the pandemic ("more of the same"), the sheer volume of such requests surpassed the support team's capacity. Consequently, there was an urgent need to recruit extra personnel. Moreover, the pandemic compelled the entire Caren team to operate remotely for the first time. To ensure the seamless flow of operations, it was crucial to strike a balance between personal and work life. |
|  | Find innovative solutions to engage the target audience. | The COVID-19 pandemic presented the Caren team with obstacles in their engagement with the target population, given that physical visits to end-users and healthcare organizations were limited. While video conferencing was available as an alternative means of involving the target group in the development process, it was not always practical, particularly for digitally inexperienced people. To address this issue, the team devised innovative solutions, such as affixing a basic iPad to a mobile robot stationed in care institutions, to establish an interactive communication channel. |
| Intervention characteristics | Automated user support is essential. | Due to an increasing number of platform users during the pandemic, automated user support became essential. In response to that, a chatbot functionality was implemented to provide automated and efficient assistance to customers (both ICs and care organizations) in resolving their issues. |
|  | Large-scale communication features are valuable. | With sudden changes in government regulations due to the fluctuating infection rates, care homes needed to adapt to the new guidelines with immediate effect. They were responsible for communicating the current guidelines and their consequences on the in-person visits between clients and their caregivers but had limited means to do so. Therefore, the functionality to send bulk messages was embedded within the Caren platform to quickly broadcast messages to all caregivers and patients at once. |
|  | Integrate care information into a single platform | Due to COVID-19, ICs’ need for digital access to reliable care-related information became even more important. However, often care information is scattered across different care organizations and not accessible via one platform. To facilitate more integrated care, the Caren team plans to provide possibilities for integrating care information stemming from different electronic client records (such as X-rays, medical history, blood test reports) in the platform. |
| Characteristics of individuals | Social isolation has a positive impact on the adoption of digital technology. | Due to Covid-19, a surge in the number of new Caren users was observed. This could be because the caregivers were not able to visit their loved ones personally, (i.e, social isolation) thus leaving not many options for them to connect to their loved ones. In this sense, the pandemic acted as a change catalyst, and caregivers went from “hesitant” to “forced” to use digital care tools like Caren (as expressed by the technology developers). |
|  | Mind the fluctuation of user groups. | Unpredictable, large-scale events such as pandemics, can impact the user pool of digital care technologies, and might result in dramatic changes in their composition. Overall the number of users increased, however it impacted the socio-demographics of the user groups (e.g. with the passing of numerous users belonging to vulnerable groups by infection or by acquiring new user groups). Specifically, mental health care organizations started adopting Caren during the pandemic, causing not only a change in the user group of Caren in terms of numerosity or sociodemographic characteristics, but also expanding their entire line of business (e.g. by becoming more “Business to Business”). |
| Outer setting | Invest in a self-sufficient and sustainable business model. | During COVID-19, new digital care platforms subsidized by the Dutch government emerged on the market. This competition was accelerated by a new governmental policy that obliged care institutions to provide clients insight into their own care data online. However, most of those new platforms lacked sustainable business models and were discontinued after the government funding ran out. This strengthened the Caren team in their choice of a business model that is independent from governmental funding, and robust against rapid market change. Specifically, Caren relies now on a business-to-business-to-consumer model where healthcare institutions purchase an electronic client record that offers Caren as an add-on. |
|  | Address concerns about data transparency in healthcare. | COVID-19 accelerated the collection of care-related data online. However, participants reported that the fear of data transparency in healthcare can form a significant barrier to implementing platforms like Caren. Although required by Dutch law, healthcare organizations are sometimes hesitant to be fully transparent about and share client data collected, as they fear that it could make it easier to trace errors back to individual caregivers. To address this, technology developers emphasized the importance of educating stakeholders about the benefits of digital data exchange within a client’s care network, such as an increased quality of care. |
